# The brain-specific RasGEF very-KIND is required for normal dendritic growth in cerebellar granule cells and proper motor coordination

**DOI:** 10.1371/journal.pone.0173175

**Published:** 2017-03-06

**Authors:** Kanehiro Hayashi, Asako Furuya, Yuriko Sakamaki, Takumi Akagi, Yo Shinoda, Tetsushi Sadakata, Tsutomu Hashikawa, Kazuki Shimizu, Haruka Minami, Yoshitake Sano, Manabu Nakayama, Teiichi Furuichi

**Affiliations:** 1 Laboratory for Molecular Neurogenesis, RIKEN Brain Science Institute, Wako, Saitama, Japan; 2 Department of Anatomy, Keio University School of Medicine, Shinjuku-ku, Tokyo, Japan; 3 Laboratory for Mental Biology, RIKEN Brain Science Institute, Wako, Saitama, Japan; 4 Research Resource Center, RIKEN Brain Science Institute, Wako, Saitama, Japan; 5 Research Center for Medical and Dental Sciences, Tokyo Medical and Dental University, Bunkyo-ku, Tokyo, Japan; 6 Department of Physiology, Nippon Medical School, Bunkyo-ku, Tokyo, Japan; 7 School of Pharmacy, Tokyo University of Pharmacy and Life Sciences, Hachioji, Tokyo, Japan; 8 Advanced Scientific Research Leaders Development Unit, Gunma University, Maebashi, Gunma, Japan; 9 Department of Applied Biological Science, Faculty of Science and Technology, Tokyo University of Science, Noda, Chiba, Japan; 10 Chromosome Engineering Team, Department of Technology Development, Kazusa DNA Research Institute, Kisarazu, Chiba, Japan; University of Florida, UNITED STATES

## Abstract

Very-KIND/Kndc1/KIAA1768 (v-KIND) is a brain-specific Ras guanine nucleotide exchange factor carrying two sets of the kinase non-catalytic C-lobe domain (KIND), and is predominantly expressed in cerebellar granule cells. Here, we report the impact of v-KIND deficiency on dendritic and synaptic growth in cerebellar granule cells in v-KIND knockout (KO) mice. Furthermore, we evaluate motor function in these animals. The gross anatomy of the cerebellum, including the cerebellar lobules, layered cerebellar cortex and densely-packed granule cell layer, in KO mice appeared normal, and was similar to wild-type (WT) mice. However, KO mice displayed an overgrowth of cerebellar granule cell dendrites, compared with WT mice, resulting in an increased number of dendrites, dendritic branches and terminals. Immunoreactivity for vGluT2 (a marker for excitatory presynapses of mossy fiber terminals) was increased in the cerebellar glomeruli of KO mice, compared with WT mice. The postsynaptic density around the terminals of mossy fibers was also increased in KO mice. Although there were no significant differences in locomotor ability between KO and WT animals in their home cages or in the open field, young adult KO mice had an increased grip strength and a tendency to exhibit better motor performance in balance-related tests compared with WT animals. Taken together, our results suggest that v-KIND is required for compact dendritic growth and proper excitatory synaptic connections in cerebellar granule cells, which are necessary for normal motor coordination and balance.

## Introduction

The cerebellum plays a central role in motor learning [1]. The cerebellar cortex possesses five major types of neurons—Purkinje cells, basket cells, stellate cells, Golgi cells and granule cells [2]. Among these, cerebellar granule cells are the sole excitatory neurons and have unique morphological features, including short branched dendrites that synapse with two inputs, excitatory terminals of afferent mossy fibers and inhibitory axons of Golgi cells, while long two-branched axons called parallel fibers extend to the molecular layer to synapse with other cerebellar cortical neurons [2,3]. Cerebellar granule cells are estimated to represent approximately half of all neurons in the brain, and their soma are densely packed in the granule cell layer. They extend an average of four short claw-like dendrites to form postsynapses around large mossy fiber terminals within the glomerular rosettes [4,5]. Furthermore, cerebellar granule cells are electrically very compact, most likely because of their short and thin dendrites, which allows them to rapidly and accurately integrate the inputs from mossy fibers [6]. Loss of these cells causes impairment in motor behavior [7,8,9, 10]. Thus, it is intriguing how cerebellar granule cells differentiate their unique dendritic structures.

Very-KIND/Kndc1/KIAA1768 (v-KIND) is a Ras guanine nucleotide exchange factor (RasGEF) containing two tandem repeats of the kinase non-catalytic C-lobe domain (KIND), which is thought to be involved in protein-protein interactions [11,12]. In mice, v-KIND is preferentially expressed in various brain regions, with cerebellar granule cells expressing relatively high levels of v-KIND, and expression is sharply increased in post-mitotic cerebellar granule cells between the postnatal first and second weeks, reaching the highest levels in the brain [13]. We previously showed that v-KIND not only activates a Ras small GTPase in the mitogen-activated protein kinase (MAPK) pathway, but that it also interacts with microtubule-associated protein (MAP) 2 through the second KIND domain (KIND2) [13,14]. We also showed that siRNA knockdown and exogenous overexpression of v-KIND respectively result in the enhancement and inhibition of MAP2-positive dendrite formation in primary cultures of cerebellar granule cells and hippocampal neurons [13]. We therefore hypothesized that v-KIND acts as a signaling molecule that negatively regulates or limits dendritic growth in cerebellar granule cells, thereby establishing their unique short dendritic morphology.

To test our hypothesis, we analyzed dendritic growth in cerebellar granule cells in v-KIND knockout (KO) mice [15]. We also examined behavioral phenotypes that are regulated by the cerebellum, including motor coordination and balance. Our findings suggest that v-KIND plays a critical role in regulating dendritic growth in cerebellar granule cells, and is required for normal cerebellar function.

## Materials and methods

### Animals

v-KIND/Kndc1/KIAA1768 KO mice [15] were obtained from the RIKEN Bio Resource Center (Tsukuba, Japan). Littermates were used as WT mice for all experiments performed. The date of the birth was defined as postnatal day (P) 0. The genotype of v-KIND KO mice was identified by PCR using the following primers: primer1, 5ʹ-CGCCTTCTATCGCCTTCTTGAC-3ʹ; primer2, 5ʹ-CATTCCAGGGTAAGCGCCATC-3ʹ; primer3, 5ʹ-GACAGATGAGCATGAGTGAGG-3ʹ. All animal experiments were approved by the Animal Care and Use Committee of RIKEN (approval number: H21-2-244(4)) and Tokyo University of Science (approval number: N15006, 7; N16008, 9). Mice were housed under a 12:12-h light–dark cycle, with the dark cycle from 20:00 to 8:00. Food and water were available *ad libitum*. All surgeries and dissections were performed under sodium pentobarbital or isoflurane anesthesia, and all efforts were made to minimize suffering.

### Nissl staining

WT and v-KIND KO mice at 8 weeks of age were deeply anesthetized and transcardially perfused with 4% paraformaldehyde (PFA) in 0.1 M phosphate buffer (PB) (pH 7.4) containing 0.2% picric acid. The brains were dissected out and postfixed in 4% PFA at 4°C for several hours. Fixed brains were sagittally sectioned at 14-μm thickness with a cryostat (CM1850, Leica Microsystems, Wetzlar, Germany). The sections were dehydrated and rehydrated in a sequence of ethanol solutions and stained in 0.1% cresyl violet solution for 30 s. Then, sections were destained and dehydrated in ethanol, and cleared in xylene. Finally, sections were mounted and observed with a microscope (Eclipse E800, Nikon, Tokyo, Japan) equipped with a cooled CCD camera (SPOT, Diagnostic Instruments Inc., Sterling Heights, MI). Digital images were processed using Adobe Photoshop 6.0 software (Adobe Systems Inc., San Jose, CA). The number of stained puncta/1,000 μm^2^ were analyzed with ImageJ.

### DiI staining and morphological analysis of cerebellar granule cells

Brains from 8-week-old v-KIND KO and WT mice were fixed with 4% PFA and cut sagittally at 300-μm thickness on a vibratome (DIK-1000, D.S.K./Dosaka EM Co., Ltd., Kyoto, Japan). Staining of the brain slices with a lipophilic dye, 1,1ʹ-dioctadecyl-3,3,3ʹ,3ʹ-tetramethylindocarbocyanine perchlorate (DiI) (Thermo Fisher Scientific, Waltham, MA) was performed according to the protocol described by Gan *et al*. [16]. Briefly, tungsten particles (Tungsten M-17, Cat. 1652267, Bio-Rad Laboratories, Hercules, CA) were coated with DiI dissolved in methylene chloride and deposited into a Tefzel tube (Bio-Rad Laboratories) using a tubing prep station (Bio-Rad Laboratories). Then, DiI-coated particles were delivered onto the brain slices using a Helios Gene Gun (Bio-Rad Laboratories) through an Isopore polycarbonate membrane filter (EMD Millipore, Billerica, MA). The slices were then kept in PBS overnight. Images of granule cells stained with DiI were acquired using a confocal microscope (FV-1000, Olympus, Tokyo, Japan). Dendrites and soma of DiI-stained granule cells were traced, and various morphological parameters, including dendrite number, length, branching and terminals, were analyzed with Neurolucida software (MBF Bioscience, Williston, VT). The average number of branches and terminals, and the average length and number of dendrites were statistically evaluated with Student’s *t*-test. Data are presented as means ± SEM.

### Antibodies

The primary antibodies used for immunohistochemistry were rabbit polyclonal anti-glutamate decarboxylase 65/67 (GAD65/67) antibody (Cat. AB1511, EMD Millipore; 1:3,000), mouse monoclonal anti-vesicular glutamate transporter 2 (vGluT2) antibody (Cat. MAB5504, EMD Millipore; 1:3,000) and rabbit polyclonal anti-GABA-A receptor α6 (GABAARα6) antibody (Cat. AB5610, EMD Millipore; 1:1,000). The secondary antibodies used for immunofluorescence staining were Alexa Fluor 488-conjugated anti-mouse IgG (Cat. A11029 or A21202; 1:1,000) and Alexa Fluor 594-conjugated anti-rabbit IgG (H+L) (Cat. A11012; 1:1,000), both from Molecular Probes (Eugene, OR).

### Immunohistochemistry

Immunohistochemical analysis was essentially performed as previously described [13]. Mice at 5 weeks of age were anesthetized with sodium pentobarbital or isoflurane and transcardially perfused with PBS, followed by 4% PFA in PB. The brains were dissected, postfixed in 4% PFA at 4°C for 1 h, and cryoprotected by immersion in 20% sucrose in PBS overnight at 4°C. After embedding in Tissue-Tek OCT compound (Sakura Finetek, Tokyo, Japan), the brains were frozen in dry ice powder and cut into 10–16-μm sagittal sections at −20°C using a cryostat (CM1850, Leica Microsystems). The sections were then air-dried for 1 h, rinsed three times in PBS, and treated with methanol at −20°C for 20 min, followed by three washes with PBS at room temperature for 10 min each. After blocking with 10% normal donkey serum (Cat. D9663, Sigma-Aldrich, St. Louis, MO) in PBS containing 0.2% Triton X-100 (PBS-T), the sections were reacted with primary antibody in PBS-T containing 5% serum at 4°C overnight, then rinsed in PBS-T and incubated with Alexa Fluor-conjugated secondary antibody in PBS-T at room temperature for 1 h, followed by PBS washes. For control sections, the primary antibody was omitted to evaluate non-specific binding of the secondary antibody. Labeled sections were mounted using Fluoromount-G (eBioscience, Santa Clara, CA). Immunoreactivity was examined using a fluorescence or light microscope (Eclipse E800, Nikon) equipped with a cooled CCD camera (SPOT, Diagnostic Instruments Inc.) or a confocal laser microscope (LSM 510 META, Carl Zeiss, Oberkochen, Germany). Digital images were processed using Adobe Photoshop 6.0 software (Adobe Systems Inc.). Immunopositive puncta/1,000 μm^2^ in random areas of lobules VI-V per each section were analyzed with ImageJ.

### Transmission electron microscopy

Sample preparation for transmission electron microscopy was performed as described previously [17,18]. Briefly, deeply anesthetized v-KIND KO and WT mice were perfused with 4% PFA/2% glutaraldehyde/0.1 M PB (pH 7.4). Serial ultra-thin sections (70-nm-thick) were stained with uranyl acetate/lead citrate. Images were obtained using a transmission electron microscope (JEM-1200EX, JEOL, Tokyo, Japan). Analyses of mossy fiber terminals and granule cell postsynapses in the cerebellum were performed with ImageJ. Data were statistically analyzed using Student’s *t*-test. Data are presented as means ± SEM.

### Behavioral tests

All mice used in the experiments below were male littermates (late adolescent to young adult [8–12 weeks of age] or mature adult [30 weeks of age]) from mated heterozygotes. The experimenter was blind to the genotype in all behavioral tests. Basic sensory functions were assessed using the startle response to sound and touch, as well as with behavioral tests, as described below and in S1 File.

#### Grip strength test

To measure forelimb grip strength, mice were made to grasp a wire handle connected to a traction apparatus (Ohara & Co., Ltd., Tokyo, Japan) with the forepaws, and were slowly pulled back by their tail. The maximum tension (in *g*) before release was recorded and normalized to body weight. Three consecutive trials were evaluated.

#### Wire hanging test

The mouse was placed on the wire cage top, which was then inverted and suspended above the home cage for 20 min, and the latency to falls was recorded. The average of two trials was evaluated.

#### Home cage activity test

Home cage activity was measured as previously described [19]. Spontaneous locomotor activity in the home cage (18 cm wide × 14 cm high × 32 cm deep) was determined by photobeam interruption with SCANET (6 channel SV-20 system, Melquest, Toyama, Japan) for 6 days after habituation to a fresh cage for 24 h. The distance between sensors was 0.5 cm. Mice were housed individually under a 12:12-h light–dark cycle, as described above. Food and water were freely available to the mice throughout the experiment. The experiment was begun at 8:00 in the light cycle, and lasted one week.

#### Open field test

The test was performed as previously described [19]. Briefly, locomotor activity was measured with an open field apparatus (60 × 60 cm) illuminated at 50 lux (at surface level). Each mouse was placed in the center of the open field, and its horizontal movements were monitored for 15 min with a CCD camera. The images were processed with NIH IMAGE O.F. software (O’Hara & Co.). The 15-min observation period was divided into three 5-min bins. Total activity in each bin was used in the statistical analysis. Rearing activities (movement in the vertical dimension) were counted for 5-min bins. Data were statistically analyzed with Student’s *t*-test. Data are presented as means ± SEM.

#### Balance beam test

The balance beam test was performed as described previously [20], with slight modification. Young adult male mice (9–10-weeks-old, +/+ and -/-) were used for the test. A 1-meter columnar beam was placed 70 cm above the floor. Mice were habituated to the test room for 1 h before starting the trials. Each mouse was placed at the origin of the beam, and a black goal box was placed at the far end of the beam. On the first day, mice were first trained to traverse a square beam (14 mm in width). On the next day, mice were tested to cross the 9-mm columnar beam 3 times, and then the 6-mm columnar beam 3 times. The fastest time to traverse the beam (up to 120 s) from three trials and the average number of slips of the hind limb off the beam before reaching the goal box were analyzed. Data were statistically analyzed with Student’s *t*-test and are presented as means ± SEM.

### Statistical analysis

Values are expressed as the mean ± SEM. Statistical analyses were performed using JMP software (SAS Institute Inc., Cary, NC) and Microsoft Excel (Redmond, WA). Normality of data distribution was confirmed by Student’s *t*-test. Statistical significance was defined as *p* < 0.05.

## Results

### Loss of v-KIND affects dendritic complexity but not the density of granule cells in the granule cell layer of the cerebellum

**Fig 1:**
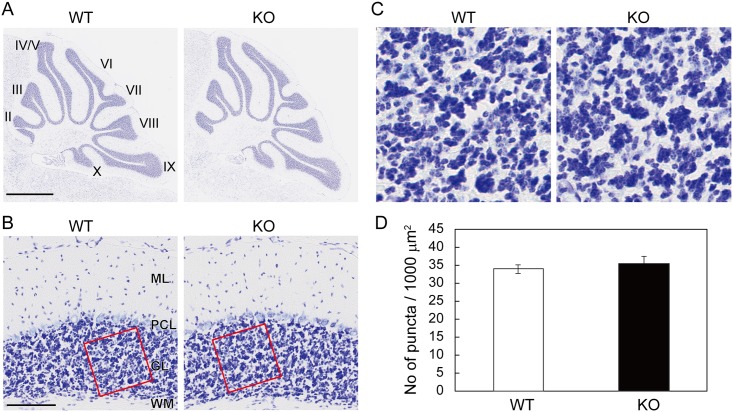
No apparent gross abnormality in the cerebellar lobules or layers, or in the densely-packed granule cell layer in Nissl-stained sections of v-KIND KO cerebella. Sagittal sections of KO and WT mice cerebella (8-week-old) were analyzed by Nissl staining. (A) The lobular structure of the whole cerebellum. Scale bar, 1 mm. (B) The layer structure of the cerebellar cortex. Scale bar, 100 μm. (C) Magnified view of the granular layer indicated by the red square in (B). Cerebellar lobules II–X. ML, molecular layer; PCL, Purkinje cell layer; GL, granular layer; WM, white matter. (D) Number of Nissl-stained puncta/1,000 μm^2^ in the granule cell layer of WT and KO mice. 5~10 random areas per section, 2 sections per animal, and three mice for each genotype (*N* = 3) was statistically analyzed. Data are shown as mean ± SEM. Two sample Student’s *t*-test assuming equal variances showed no statistical significance (*p* > 0.5).

v-KIND KO mice did not exhibit any obvious abnormality in body shape, reproductive capacity or longevity, as previously reported [15]. We compared the anatomy of Nissl-stained cerebella between KO mice and their WT littermates (Fig 1). There seemed to be no difference in the number and overall appearance of the cerebellar lobules (Fig 1A) or cortical layer structure (Fig 1B) between the two genotypes. In the granule cell layer (Fig 1C), Nissl-positive puncta and Nissl-negative areas respectively correspond to the densely-packed granule cell somata and the glomeruli where granule cell dendrites are innervated by mossy fiber afferents as well as Golgi cell axons. Nissl-positive and Nissl-negative staining patterns appeared similar between the two genotypes (Fig 1C). The number of Nissl-positive puncta in KO mice were not different from those in WT mice (WT: 33.84 ± 1.21 puncta/1,000 μm^2^; KO: 35.39 ± 2.01 puncta/1,000 μm^2^; *p* = 0.523) (Fig 1D), suggesting that cerebellar granule cell density is unaffected by v-KIND deficiency.

**Fig 2:**
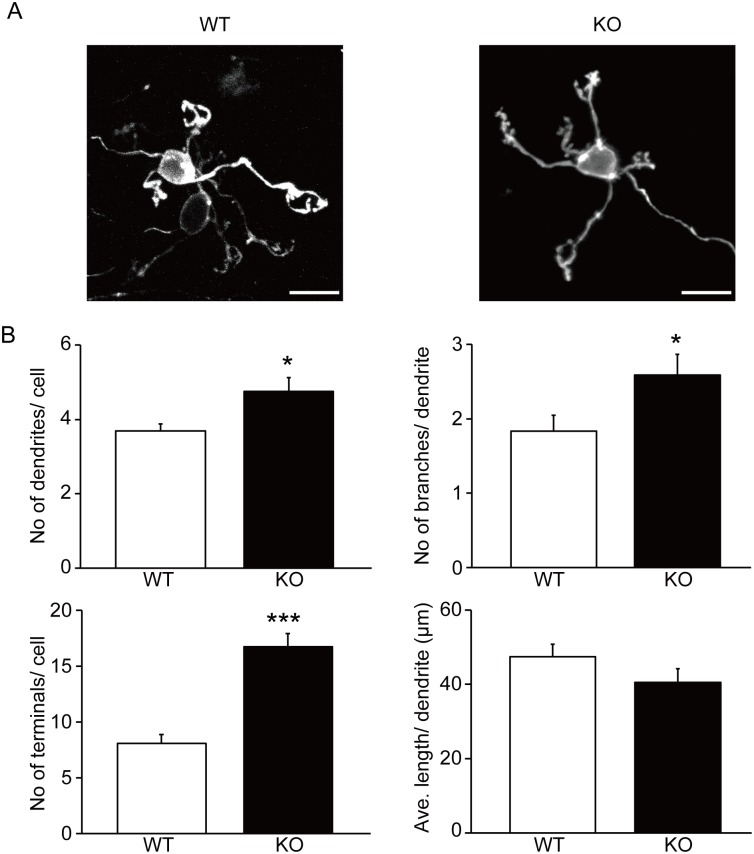
Increased branches and terminals of cerebellar granule cell dendrites in v-KIND KO cerebella. (A) Branched arborization pattern of granule cell dendrites in the granular layer of WT (*left*) and v-KIND KO (*right*) mouse cerebella at 8 weeks of age. Granule cells were visualized by DiI staining, and images were obtained by confocal microscopy. Scale bar, 10 μm. (B) Statistical analysis of the number of dendrites per cell (*top left*), number of branches per dendrite (*top right*), number of terminals per cell (*bottom left*) and average length of dendrites (*bottom right*) of cerebellar granule cells by Student’s *t*-test. Cells from four animals were analyzed for each genotype (*N* = 4) (WT: *n* = 13 cells; KO: *n* = 12 cells). Data are shown as mean ± SEM. Two sample Student’s *t*-test assuming equal variances; *p < 0.05, ***p < 0.001.

To examine dendritic growth in cerebellar granule cells in greater detail, we visualized the dendrites by staining with a fluorescent lipophilic cationic indocarbocyanine dye, DiI (Fig 2A). KO mice showed an increased number of dendrites per cell (WT: 3.69 ± 0.17; KO: 4.75 ± 1.29; *p* = 0.020), branches per dendrite (WT: 1.83 ± 0.21; KO: 2.59 ± 0.28; *p* = 0.035) and terminals per cell (WT: 8.08 ± 0.79; KO: 16.75 ± 1.19; *p* = 0.000074) compared to WT mice (Fig 2B). However, the average length of dendrites was not different between the two genotypes (WT: 47.36 ± 3.47 μm; KO: 40.52 ± 3.61 μm; *p* = 0.17). These results suggest that while KO mice do not have a severe abnormality in the gross structure of the cerebellum, there is an overgrowth of granule cell dendrites, leading to an increased number of dendrites, branches and terminals.

### v-KIND KO mice have aberrant excitatory synaptic organization in the cerebellar glomeruli

**Fig 3:**
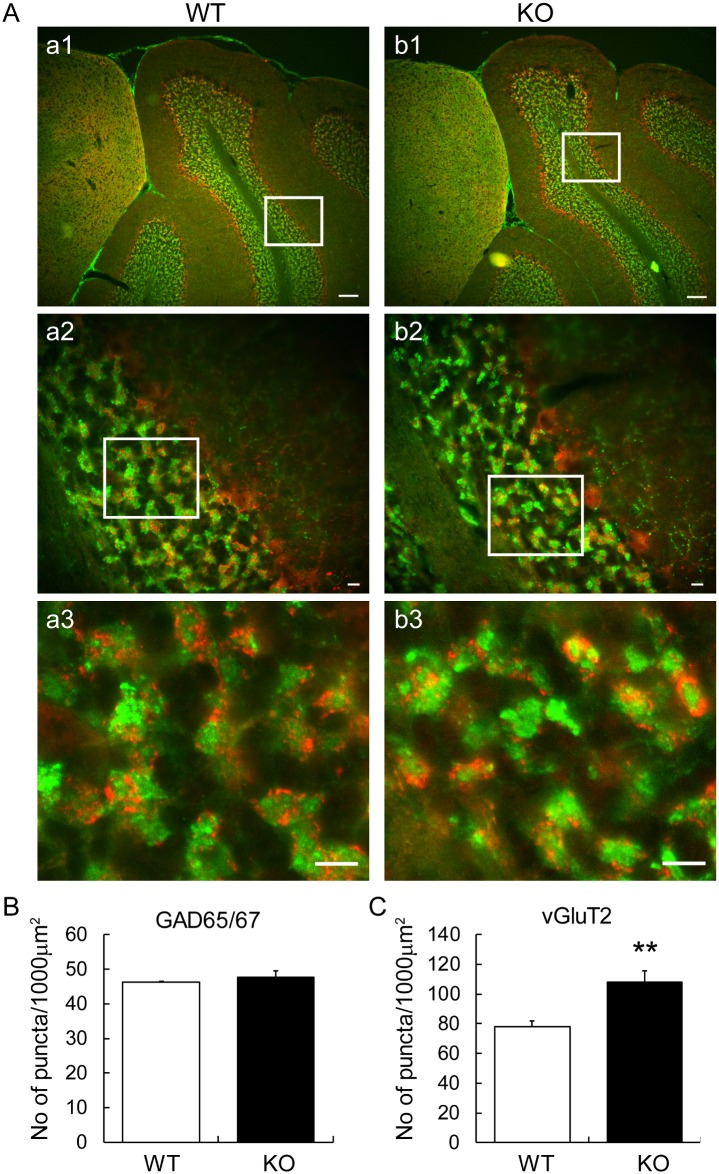
Expression of presynaptic markers for mossy fibers and Golgi cell axons in cerebellar glomeruli of v-KIND KO mice. (A) Immunohistochemistry of the presynaptic GAD65/67 protein (red) in Golgi cells and the presynaptic vGluT2 (green) in mossy fibers in the granule cell layers of WT and KO mice at 5 weeks of age. Panels a1 and b1 show representative images of co-immunostaining patterns in lobules IV–V of WT and KO mice, respectively. Panels a2 and b2 are magnified images of the cerebellar layer indicated by the white square in a1 and b1, respectively. Panels a3 and b3 are magnified images of the granular layer indicated by the red square in a2 and b2, respectively. Bars: 100 μm in a1 and b1, 10 μm in a2, a3, b2 and b3. Images of vGluT2 immunostaining of whole cerebellar sections are shown in S1 Fig. (B) Number of GAD65/67-immunopositive puncta (/1,000 μm^2^) was calculated by analyzing data from fifteen areas (7,182 μm^2^/area) (*n* = 15) of three different animals (*N* = 3) for each genotype. (C) Number of vGluT2-immunopositive puncta (/1,000 μm^2^) was calculated by analyzing 26 and 24 areas (2,500 μm^2^/area) in WT (*n* = 26) and KO (*n* = 24) mice, respectively, from five different animals (*N* = 5) for each genotype. Data are shown as mean ± SEM. Two sample Student’s *t*-test assuming equal variances (GAD65/67 *N* = 3; vGluT2 *N* = 5); ** *p* < 0.01 in (C).

To investigate the synaptic organization of granule cells in KO mice, we examined cerebellar glomeruli where granule cell dendrites receive excitatory and inhibitory inputs from mossy fibers and Golgi cell axons, respectively, in lobule VI/V of sagittal sections by immunohistochemistry using anti-GAD65/67 and vGluT2 antibodies (Fig 3A: a1–a3, WT; b1–b3, KO). The number of GAD65/67-immunopositive puncta in inhibitory Golgi cell axons was not different between the two genotypes (WT: 46.3 ± 0.1 puncta/1,000 μm^2^; KO: 47.7 ± 1.8 puncta/1,000 μm^2^; *p* = 0.505) (Fig 3B). The number of vGluT2-immunopositive puncta in excitatory mossy fibers was increased in KO mice (107.9 ± 7.5 puncta/1,000 μm^2^) compared to the WT (78.0 ± 3.9 puncta/1,000 μmm^2^) (*p* = 0.0076) (Fig 3C). The overall immunostaining patterns of vGluT2 (S1 Fig) and GABAAR-α6 (S2 Fig) in cerebellar lobules IV–V seemed similar between WT and KO mice, suggesting no difference in the molecular compartmentation around the lobules of almost the same mediolateral and anteroposterior sections between WT and KO mice.

**Fig 4:**
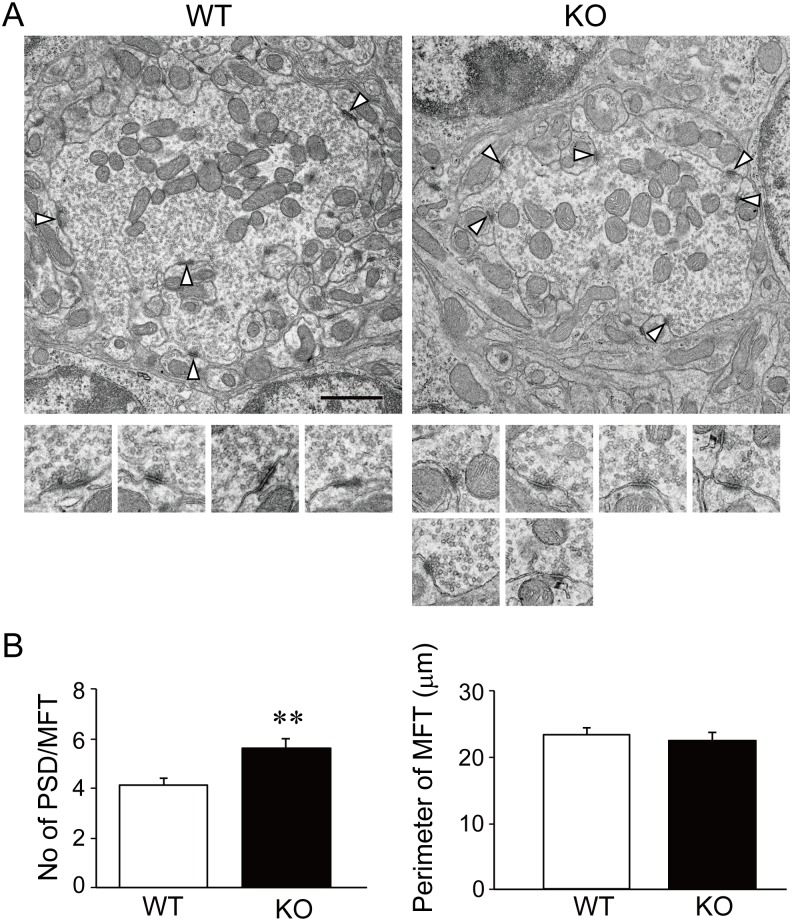
Increased number of postsynaptic densities in mossy fiber–granule cell synapses in v-KIND KO cerebellum. (A) Representative electron microscopic images of cerebellar glomeruli in WT (*left*) and v-KIND KO (*right*) mice (8-week-old). Arrow heads indicate excitatory synapses between mossy fiber terminals (MFTs) and dendrites of cerebellar granule cells. High power views of each synaptic structure indicated by arrow heads are shown below. Scale bar, 1 μm. (B) Structural analysis of granule cell–mossy fiber synapses in the glomerulus. *Left*, the number of granule cell postsynaptic densities (PSDs) per MFT was increased in the KO compared with WT. *Right*, the perimeter of the MFT was not different between KO and WT animals. MFTs in two different electron microscopic images from 3 independent mice (*N* = 3) for each genotype were analyzed (number of MFTs analyzed: WT, *n* = 50; KO, *n* = 53). Data are shown as mean ± SEM. Two sample Student’s *t*-test assuming equal variances; **p < 0.01.

We next examined the ultrastructure of the cerebellar glomeruli in WT and KO mice by electron microscopy (Fig 4A). KO mice displayed an increase in the number of postsynaptic densities in granule cells per mossy fiber terminal (5.60 ± 0.38) compared to WT mice (4.12 ± 0.30) (*p* = 0.003), although the size of the mossy fiber terminal was not different between the two genotypes (WT: 23.27 ± 1.13 μm; KO: 22.42 ± 1.24 μm; *p* = 0.61). The area of the mossy fiber terminal and the width of the postsynaptic density were unchanged (data not shown). Taken together, these results suggest that the overgrowth of granule cell dendrites in KO mice may influence the organization of mossy fiber–granule cell synapses within the cerebellar glomeruli.

### v-KIND KO mice exhibit normal gross motor skills, but show a tendency for increased strength and enhanced motor coordination

**Fig 5:**
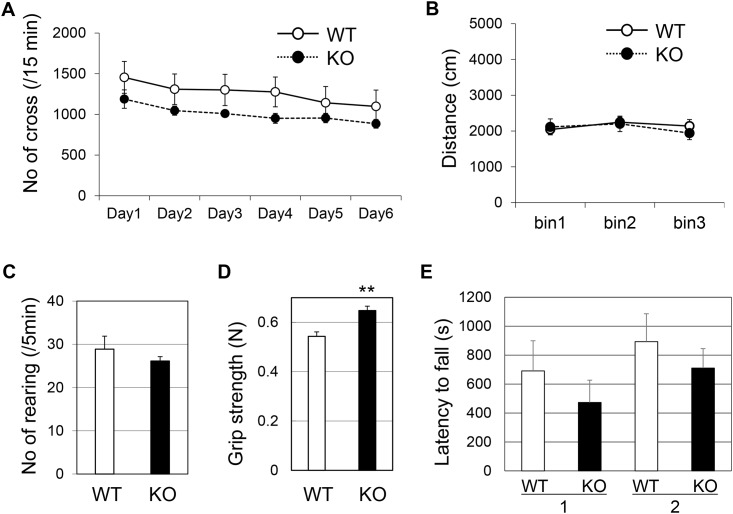
v-KIND KO mice show increased grip strength but no difference in locomotion or wire hanging ability compared to their WT littermates. Male mice (8–12 weeks of age) were tested. (A) Home cage activity (indicated by the number of crossings of the beam per 15 min) during the dark period for 6 days. WT: *n* = 9; KO: *n* = 8. There was no statistically significant difference between the two genotypes under dark (Fig 5A) or light (data not shown) conditions. (B) Distance (cm) traveled in an open field, illuminated at 50 lux, in 3 5-min bins. WT: n = 10; KO: n = 10. (C) Number of rearings in a period of 5 min. WT: *n* = 9; KO: *n* = 8. (D) Grip strength (in Newton [N]). WT: *n* = 6; KO: *n* = 10. (E) Latency to fall (s) in three trials of wire hanging is shown. WT: *n* = 6; KO: *n* = 10. Data are shown as mean ± SEM. Two sample Student’s *t*-test assuming equal variances; **p < 0.01 in (D).

We next analyzed the motor phenotype of KO mice. Male v-KIND KO mice showed no change in body weight (WT: 25.57 ± 0.43 g; KO: 25.55 ± 0.55 g; *p* = 0.98), and they appeared to have normal basic sensory functions (visual, auditory, olfactory and somatosensory) compared to their WT littermates (S1 Table). There was no difference in locomotor ability in the home cage (Student’s *t*-test genotype: *p* = 0.16) (Fig 5A) or in the open field (genotype: *p* = 0.81) (Fig 5B). Similarly, there was no difference in rearing activity (number of rearings/5 min: WT: 28.89 ± 2.99; KO: 26.13 ± 1.03; *p* = 0.40) (Fig 5C) between the two genotypes. Interestingly, KO mice showed an increase in grip strength compared to WT animals (WT: 0.54 ± 0.02 N; KO: 0.65 ± 0.02 N; *p* = 0.0017) (Fig 5D). However, this did not contribute to better performance in the wire hanging test ((latency to fall [s]) WT: 690.83 ± 208.74; KO: 472.80 ± 154.10; *p* = 0.42 in the first trial; WT: 893.33 ± 192.40; KO: 710.50 ± 134.53; *p* = 0.45 in the second trial) (Fig 5E).

**Fig 6:**
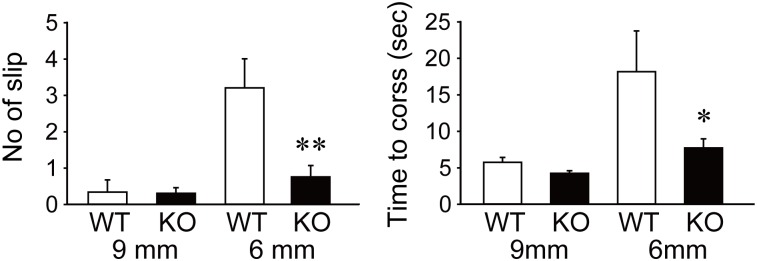
v-KIND KO mice have a tendency to display better motor performance than WT mice. Balance beam test of WT and v-KIND KO mice. *Left*, number of slips while the mouse remained on the 9-mm or 6-mm beam. *Right*, time (s) the mouse crossed the 9-mm or 6-mm beam. Data are shown as mean ± SEM (WT: *n* = 6; KO: *n* = 10). *p < 0.05, **p < 0.01 (two-tailed, unequal variances Student’s *t*-test).

We further examined the locomotor performance of 8-week-old KO and WT mice (Fig 6). In the balance beam test, both WT and KO mice walked steadily on the 9-mm beam and only rarely slipped off ((time to cross [s]) WT: 5.7 ± 1.5; KO: 4.2 ± 1.1; *p* = 0.10). When the beam was replaced with a narrower one (6-mm), it took much longer for WT mice to cross over to the other side, and they occasionally slipped. In comparison, KO mice walked smoothly on the narrower beam, with a shorter time to cross (WT: 18.1 ± 12.5 s; KO: 7.7 ± 3.6 s; *p* = 0.04) and fewer slips (WT: 3.2 ± 1.9 slips; KO: 0.75 ± 0.9 slips; *p* = 0.007) (Fig 6). In the rotarod performance test, KO mice at 8 weeks of age exhibited a tendency to show slightly better performance in motor coordination skill training on only the first trial on each day compared with 8-week-old WT mice (S3 Fig). However, WT and KO mice showed a similar score after motor skill learning. Older mature adult v-KIND KO mice at 30 weeks of age did not exhibit superior performance on the same test compared with WT mice (S3 Fig). The results suggest that young adult KO mice (8-week-old) have a subtly higher motor coordination skill in the balance beam test but not rotarod test. KO mice showed no significant changes in various behavioral tests for memory, cognition, emotion and nociception compared with WT mice (S1 Table).

## Discussion

Our findings indicate that v-KIND deficiency alters dendritic structure and excitatory synaptic connections in cerebellar granule cells. Furthermore, these changes are probably associated with increased grip strength and better motor coordination, although the gross anatomy of the cerebellum as well as basic motor phenotypes appear normal. These results suggest that v-KIND is required for normal, compact, dendritic growth in cerebellar granule cells, which is necessary for proper motor coordination and balance.

Expression of v-KIND protein seems to be strictly regulated in cerebellar granule cells during the postnatal stage in the mouse cerebellum [13]. v-KIND is predominantly expressed in post-mitotic granule cells that reach the internal granular layer after migrating from the external granular layer. Its expression suddenly becomes apparent between the first and second postnatal weeks and increases to peak around the weaning period and thereafter, coincident with the stage of dendritic growth and synaptic maturation in cerebellar granule cells. Although v-KIND KO mice have normal patterns of cerebellar lobulation and cortical layer structure, they display an increased number of dendrites and branches in cerebellar granule cells, resulting in a greater number of synapses per mossy fiber terminal. The number, but not length, of granule cell dendrites is decreased by expression of dominant-negative erbB4, suggesting the involvement of neuregulin/erbB signaling in dendrite formation [21]. v-KIND, which has RasGEF activity [13], may regulate the Ras-MAPK signaling pathway downstream of erbB. Taken together, these results suggest that v-KIND is involved in the fine tuning of dendritic and synaptic development in cerebellar granule cells, the largest cell population in the mammalian brain.

v-KIND KO mice are unique in exhibiting a slight enhancement in grip strength and balance skill; most cerebellar mutants perform poorly in similar motor tests, including mice with mutations affecting granule cell synapses with excitatory mossy fibers [7] and inhibitory Golgi cells [8], as well as mutants with perturbed granule cell proliferation and maturation [9]. This raises the question of whether the enhanced performance is caused by the increased number of dendritic branches and synapses in cerebellar granule cells. The acute ablation of cerebellar Golgi cells disrupts the balance between inhibitory and excitatory transmission in cerebellar glomeruli, resulting in overexcitation and impaired motor coordination, which gradually partially recovers by the functional attenuation of NMDA receptors [8]. Thus, a change in the number of excitatory mossy fiber–granule cell synapses in KO mice may not simply contribute to better motor skill. Interestingly, better motor balance was observed in early adulthood but yet unclear in mature adult stage. The accelerated dendritic development and synapse formation in v-KIND KO mice may result in the earlier maturation of motor ability. In this context, it is worth noting that P28, but not P56, CAPS2 KO mice showed decreased paired-pulse facilitation at parallel fiber-Purkinje cell synapses as well as rotarod performance compared with WT mice [17] and that old, but not middle-aged, tau KO mice exhibit impairment in hippocampal synaptic function and a deficit in the Morris water maze test (likely caused by an instability of MAPs) [22], suggesting a postnatal stage-related phenotype which is getting conspicuous, better or worth. Further study is needed to clarify v-KIND function by analyzing the link between dendritic overgrowth and motor skills, as well as the postnatal stage or sate-related phenotypes in different types of behavior. In addition, since there is the possibility that the effect observed in the present study might be due to a global v-KIND KO, a cerebellum-specific KO will be worth pursuing.

The immunoreactivities for vGluT2 and GAD65/67 shown in Fig 3 are mostly derived from mossy fiber and Golgi axon terminals, respectively, innervating granule cells, the majority cell population in the granular layer, although there are small populations of excitatory neurons (e.g., unipolar brush cells and Lugaro cells) in this layer as well. Our results show that the absence of v-KIND function not only increases the number of postsynaptic densities in granule cell dendrites connecting a mossy fiber terminal, it also produces an increase in the number of vGluT2-positive puncta (probably reflecting mossy fiber terminals), suggesting that the alteration in granule cell dendrites may influence mossy fiber terminals in the glomerular rosettes. Cerebellar granule cells secrete a synaptogenic factor, Wnt-7a, that induces axonal remodeling in mossy fibers and the maturation of glomerular rosettes [23]. A recent *in vivo* imaging study found that mossy fiber axons and terminals are structurally stable over a period of 2–3 weeks postnatally [24]. v-KIND deficiency in cerebellar granule cells may cause ultrafine, but not robust, rearrangement in mossy fibers within narrow glomerular spaces densely occupied by granule cell soma in the granule cell layer. Interestingly, the time window of Wnt-7a expression is similar to that of v-KIND during the postnatal stage—Wnt-7a is only expressed at low levels in the first postnatal week and reaches a peak between the second and third postnatal weeks. In addition, both Wnt-7a and v-KIND regulate microtubule dynamics through the phosphorylation of microtubule-binding protein [13,14,23]. Future studies should clarify the molecular mechanisms underlying the v-KIND-dependent regulation of compact dendrite formation in cerebellar granule cells. For example, the crosstalk between the Wnt-7a and v-KIND pathways can be investigated to determine whether they interact to establish the compact dendritic structure in these cells.

In conclusion, our findings suggest that v-KIND is required for proper dendritic growth and the formation of excitatory synaptic connections in cerebellar granule cells, which are needed for normal motor coordination and balance, at least in young adults.

## Supporting information

S1 Fig vGluT2-immunoreactivity in whole cerebellar sections of wild-type and v-KIND KO mice. (TIF)

S2 Fig GABAAR-α6 immunoreactivity in cerebellar lobules IV–V of wild-type and v-KIND KO mice. (TIF)

S3 Fig Rotarod performance test. (TIF)

S1 File Materials and methods. (DOCX)

S1 Table Summary of behavioral tests concerning memory, cognition, emotion and nociception. (DOCX)
