# Supplementary figures and images for: The brain-specific RasGEF very-KIND is required for normal dendritic growth in cerebellar granule cells and proper motor coordination
Source: PLoS One. 2017 Mar 6;12(3):e0173175. doi: 10.1371/journal.pone.0173175 (PMC5338823; doi:10.1371/journal.pone.0173175)

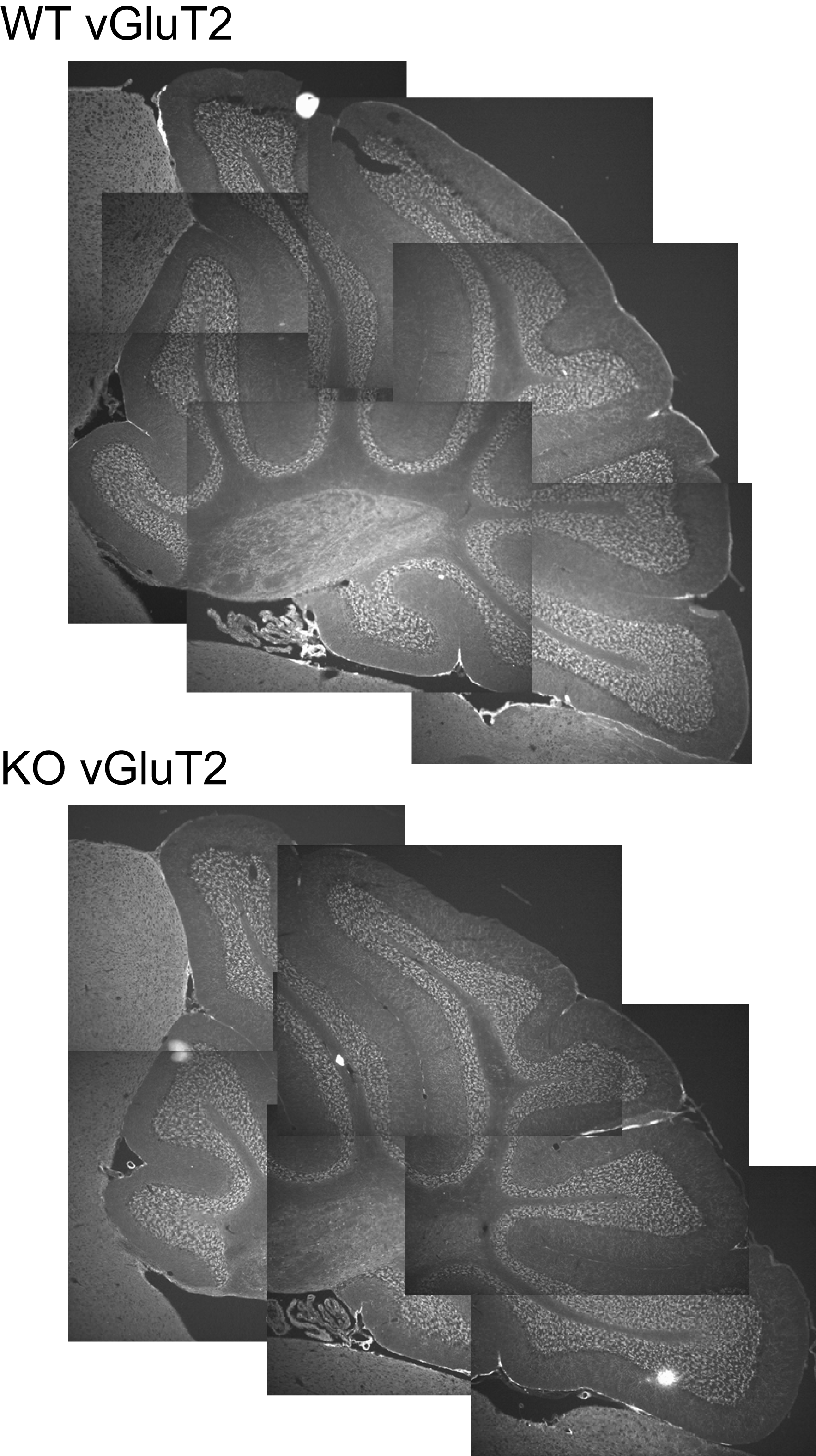

Supplement: S1 Fig — (TIF) [file pone.0173175.s001.tif]

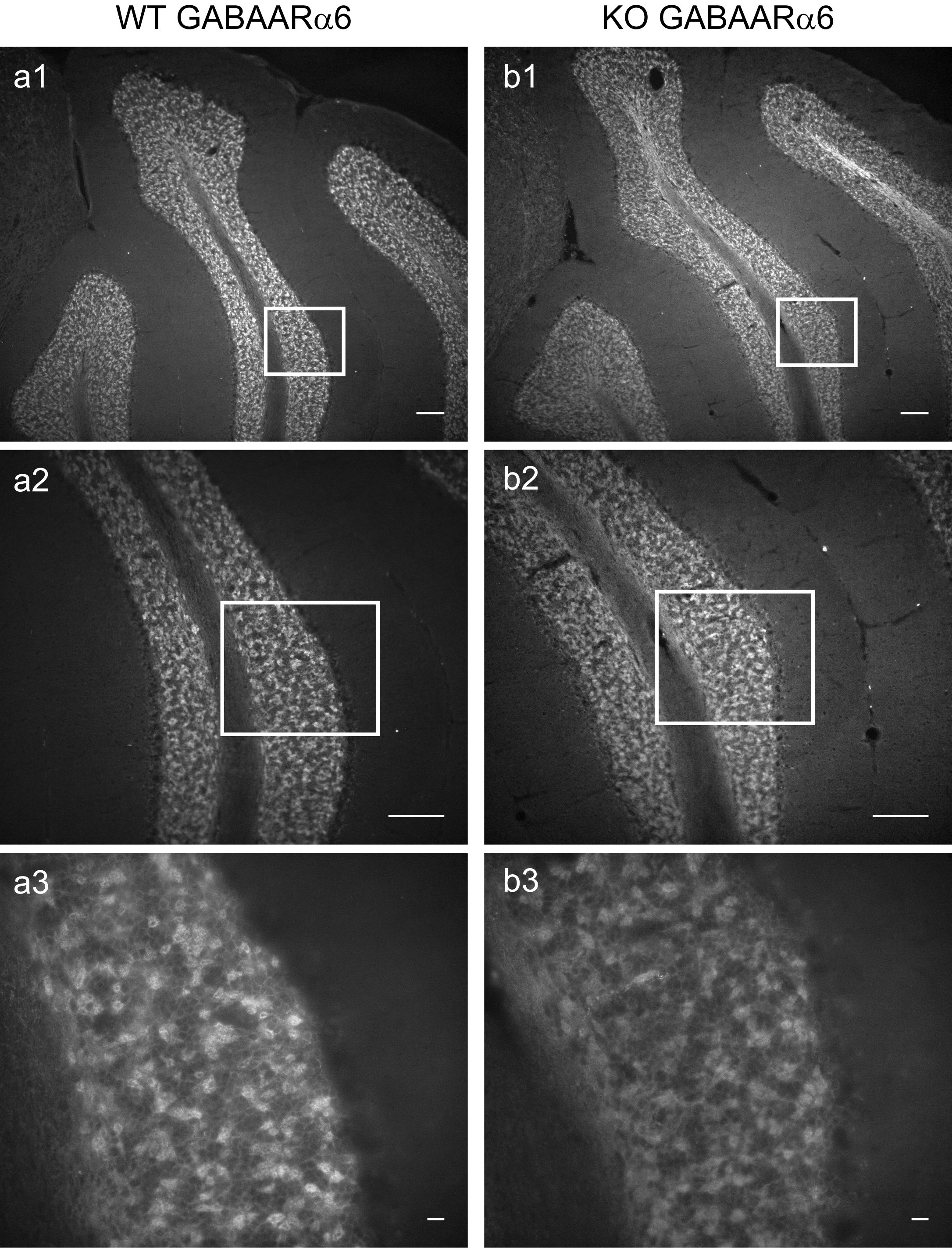

Supplement: S2 Fig — (TIF) [file pone.0173175.s002.tif]

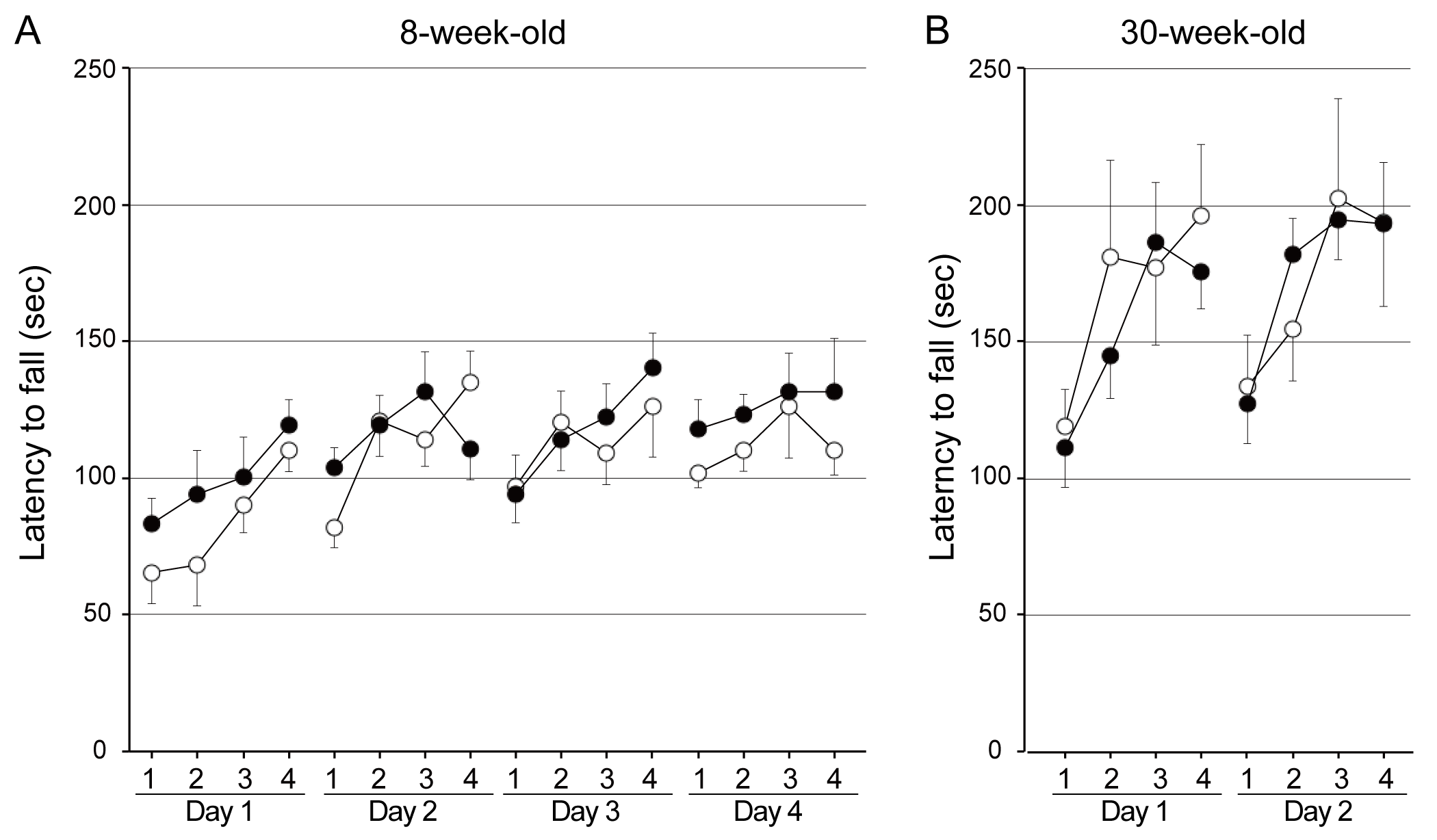

Supplement: S3 Fig — (TIF) [file pone.0173175.s003.tif]
