# Supplementary material for: The brain-specific RasGEF very-KIND is required for normal dendritic growth in cerebellar granule cells and proper motor coordination
Source: PLoS One. 2017 Mar 6;12(3):e0173175. doi: 10.1371/journal.pone.0173175 (PMC5338823; doi:10.1371/journal.pone.0173175)
Supplement: S1 File — (DOCX) [file pone.0173175.s004.docx]

**Supporting Information**

**Materials and Methods**

**Behavioral tests**

All experimental protocols were approved by the RIKEN Institutional Animal Care and Use Committee. All mice used in the experiments below were male littermates from mated heterozygotes. The experimenter was blind to the genotype in all tests. The mice were housed under a 12-h light/dark cycle, with the dark cycle from 8:00 P.M. to 8:00 A.M.

**Open field test/Novelty recognition test**

Open field tests, with or without a novelty object, were performed as previously described (Sadakata et al., 2007, Shinoda et al., 2011). Two-month-old male mice were used for the open field test (WT: *n* = 9; KO: *n* = 8). Four-month-old male mice were used for the novelty recognition test (WT: *n* = 10; KO: *n* = 10).

**Light-Dark box test**

The test was performed as previously described (Sadakata et al., 2007). Two-month-old male mice were used for the test (WT: *n* = 9; KO: *n* = 8).

**Elevated plus maze test**

The test was performed as previously described (Shinoda et al., 2011). Three-month-old male mice were used for the test (WT: *n* = 9; KO: *n* = 8).

**Acoustic startle response/Pre-pulse inhibition (PPI) test**

The tests were performed as previously described (Matsumoto et al., 2011). Three-month-old male mice were used for the tests (WT: *n* = 9; KO: *n* = 8).

**Morris water maze test**

The test was performed as previously described (Shinoda et al., 2011). Four-month-old male mice were used for the test (WT: *n* = 9; KO: *n* = 8).

**Contextual fear conditioning test**

The test was performed as previously described (Shinoda et al., 2011). Four-month-old male mice were used for the test (WT: *n* = 9; KO: *n* = 8).

**Social interaction test:**

The test was performed as previously described (Sadakata et al., 2007). Two-month-old male mice were used for the test (WT: *n* = 10; KO: *n* = 10).

**Y-maze test**

The test was performed as previously described (Shinoda et al., 2011). Three-month-old male mice were used for the test (WT: *n* = 10; KO: *n* = 10).

**Barnes maze test**

The test was performed as previously described (Shinoda et al., 2011). Three-month-old male mice were used for the test (WT: *n* = 10; KO: *n* = 10).

**Tail suspension test**

The test was performed as previously described (Shinoda et al., 2011). Three-month-old male mice were used for the test (WT: *n* = 10; KO: *n* = 10).

**Hot plate test**

Three-month-old male mice were used for the test (WT: *n* = 10; KO: *n* = 10). Each mouse was placed on a pre-heated plate (52 °C). The latencies to licking of the paws and flinching were recorded. The cut-off time was 90 s.

**Passive avoidance test**

Four-month-old male mice were used for the test (WT: *n* = 10; KO: *n* = 10). The apparatus consisted of two rooms, a light compartment and a dark compartment, which were separated by a mobile shutter (O’Hara & Co.). On the first day (conditioning day), each mouse was placed into the light room. Then, 60 s later, the shutter was opened. When the mouse entered the dark room, an electric shock (0.3 mA for 1s) was delivered through the grid floor. On the next day, each mouse was again placed into the light room, and the latency to enter the dark room was recorded. The cut-off time was 300 s.

**Rotarod test**

The rotarod test was performed as described previously (Sadakata et al., 2007) using Rota-Rod Treadmills (Model MK-610A, Muromachi Kikai Co., Ltd., Tokyo, Japan). The same male WT (*n* = 6) and v-KIND KO (*n* = 10) mouse groups were used at either 8 weeks (for young adult) or 30 weeks (for mature adult) of age. Mice were habituated to the test room for 1 h before the trial. On the first day, each mouse was placed on the stationary rod (0 rpm) for four successive trials, followed by four trials at a rotation speed of 4 rpm. Latency to a fall was monitored for 120 s. Inter-trial intervals for each animal were greater than 20 min. On the first day, after habituation to the room, each mouse was placed on the rotarod rotating at 4 rpm, and then, the rotation of the rod was accelerated from 4 to 50 rpm over a period of 180 s at a constant rate. The rotation speed of 50 rpm was subsequently maintained for 60 s. The time each mouse was able to remain on the rod was measured. Mice were trained for 4 (8-week-old) or 2 (30-week-old) consecutive days and underwent four trials per day, with an interval of 10 min between trials. Data were statistically analyzed with ANOVA followed by Tukey-Kramer’s test.

**Statistics**

Values are expressed as the mean ± SEM. Statistical analyses were performed using JMP software (SAS Institute Inc., Cary, NC) and Microsoft Excel (Redmond, WA). Data were statistically analyzed with the 2-tailed Student’s *t*-test or the Mann-Whitney *U*-test, according to homoscedasticity or heteroscedasticity, respectively. Statistical significance was defined as *p* < 0.05.

**References**

Matsumoto Y, Katayama K, Okamoto T, Yamada K, Takashima N, Nagao S, and Aruga J. Impaired auditory-vestibular functions and behavioral abnormalities of Slitrk6-　deficient mice. PLoS One. 2011; 6: 1–10.

Sadakata T, Washida M, Iwayama Y, Shoji S, Sato Y, Ohkura T, et al. Autistic-like phenotypes in Cadps2-knockout mice and aberrant CADPS2 splicing in autistic patients. J Clinc Invest. 2007; 117: 931-943.

Sadakata T, Kakegawa W, Mizoguchi A, Washida M, Katoh-Semba R, Shutoh F, et al. Impaired cerebellar development and function in mice lacking CAPS2, a protein involved in neurotrophin release. J Neurosci. 2007; 27: 2472–2482.

Shinoda Y, Sadakata T, Nakao K, Katoh-Semba R, Kinameri E, Furuya A, et al. Calcium-dependent activator protein for secretion 2 (CAPS2) promotes BDNF secretion and is critical for the development of GABAergic interneuron network. Proc Natl Acad Sci USA. 2011; 108: 373–378.

**Figure legends**

**S1 Fig. vGluT2-immunoreactivity in whole cerebellar sections of wild-type and v-KIND KO mice.** Cerebellar sections of WT (top panel) and KO (bottom panel) mice at 5 weeks of age were immunostained for vGluT2.

**S2 Fig. GABAAR-α6 immunoreactivity in cerebellar lobules IV–V of wild-type and v-KIND KO mice.** Cerebellar lobules IV–V of WT (a1–a3) and KO (b1–b3) mice at 5 weeks of age were immunostained for GABAAR-α6. Panels a2, a3, b2 and b3 are the enlarged views of the regions indicated by the white squares in panels a1, a2, b1 and b2, respectively. Scale bars, 100 μm in a1 and b1; 10 μm in a2, a3, b2 and b3.

**S3 Fig. Rotarod performance test.** The rotarod test was conducted on 8-week-old (A) or 30-week old (B) WT (open circles) and v-KIND KO (filled circles) mice. The mouse was placed on a rotarod rotating at 4 rpm, and then, the rotation speed of the rod was increased to 50 rpm. The latency (s) to falling off the rod was measured in four trials per day with a 1-h interval between trials. The test was conducted for 4 consecutive days. Each plot shows means ± SEM for WT (*n* = 6) and KO (*n* = 10) mice at 8 weeks of age and WT (*n* =6) and KO (*n* =10) mice at 30 weeks of age.

**List Supporting Information captions**

**S1 Fig. vGluT2-immunoreactivity in whole cerebellar sections of wild-type and v-KIND KO mice.**

**S2 Fig. GABAAR-α6 immunoreactivity in cerebellar lobules IV–V of wild-type and v-KIND KO mice.**

**S3 Fig. Rotarod performance test.**

**S1 Table. Summary of behavioral tests concerning memory, cognition, emotion and nociception.**
