# Supplementary material for: The brain-specific RasGEF very-KIND is required for normal dendritic growth in cerebellar granule cells and proper motor coordination
Source: PLoS One. 2017 Mar 6;12(3):e0173175. doi: 10.1371/journal.pone.0173175 (PMC5338823; doi:10.1371/journal.pone.0173175)
Supplement: S1 Table — (DOCX) [file pone.0173175.s005.docx]

**S1 Table. Summary of behavioral tests concerning memory, cognition, emotion and nociception**

WT KO *p* value

Open field test (Time in center, sec) 172.11 ± 15.35 202.81 ± 41.78 *p* = 0.082

Light-Dark box test (% time in light chamber) 42.73 ± 1.59 42.92 ± 2.11 *p* = 1.000

Elevated plus maze test (% time in open arm) 9.34 ± 3.86 17.31 ± 4.34 *p* = 0.160

Water maze test (% in target) 34.81 ± 3.29 27.71 ± 2.50 *p* = 0.139

Fear conditioning test (% freezing) 44.22 ± 5.41 46.93 ± 10.25 *p* = 0.545

Y-Maze test (% alternation) 59.51 ± 3.41 54.78 ± 4.09 *p* = 0.280

Barnes maze test (latency to enter) 35.24 ± 3.38 35.45 ± 3.26 *p* = 0.970

Passive avoidance test (sec) 188.20 ± 45.06 179.20 ± 41.89 *p* = 0.904

Social Interaction (No of participate) 1.50 ± 0.019 1.51 ± 0.046 *p* = 0.850

Novel object recognition test (% on novelty) 10.58 ± 1.99 9.56 ± 1.73 *p* = 0.650

Startle response (mean score, AU) 1.84 ± 0.14 2.08 ± 0.24 *p* = 0.420

PPI test (inhibition ration, %) 48.28 ± 4.00 31.02 ± 8.25 *p* = 0.089

Tail Suspension test (% in immobility) 37.86 ± 6.28 33.5 ± 4.62 *p* = 0.650

Hot plate test (Lick) (sec) 13.98 ± 0.88 16.09 ± 1.94 *p* = 0.156

Hot plate test (Flinch) (sec) 18.42 ± 1.24 17.87 ± 2.28 *p* = 0.196

Values are expressed as the mean ± SEM. AU, arbitrary unit
